# Clinical Lecture on Scarlet Fever

**Published:** 1870-04

**Authors:** William Jenner

**Affiliations:** Physician in Ordinary to H.M. the Queen; Professor of Clinical Medicine in University College, and Physician to the Hospital


### CLINICAL LECTURE ON SCARLET FEVER.

By Sir WILLIAM JENNER, Bart., M.D., F.R.S., Physician in Ordinary to H.M. the Queen; Professor of Clinical Medicine in University College, and Physician to the Hospital.

Gentlemen:—Cases of scarlet fever are not usually admitted into the wards. There is, in fact, a rule of the hospital by which they are excluded, on account of the disease being contagious. But it happened, not long ago, that some cases were admitted by accident, and one child sickened of the disease while in the hospital. I shall take this opportunity of bringing those cases under your notice, together with some others, which will, I hope, impress upon your mind all the most important points to be remembered in regard to this very common disease.

The contagious diseases of this class will form a large proportion of the acute diseases you will see in your after-life, and it is unfortunate that the great hospitals afford you no opportunity for studying them clinically. And this consideration makes me especially desirous of lecturing to you on these cases.

To-day, I shall refer to cases in which the scarlet fever was free from complication, though the general disease—the scarlet fever—ranged in degree from the most trifling affection to one grave enough to kill. The disease in all these cases, however, was scarlet fever, pure and simple, the throat affection even being trifling in degree. I will first read to you the abstracts of some cases.

A child eight years old was in the hospital, when, on Sunday, Nov. 15th, she complained of not feeling well, and was feverish; her skin, that is to say, felt hot, and her throat was sore. In the evening of the same day, her neck and face were very red, and on the next day, Monday, she was covered with the scarlatina rash. The tonsils were then bright red and swollen. Her temperature was 101.2°, three degrees higher than in health. On the Wednesday, the rash was almost confined to the trunk and legs—*i. e.*, it had faded on the upper part of the trunk, and the upper extremities, and had spread downwards to the legs. Her tonsils were still swollen, though no longer red; her temperature was 100°. On the Friday, the rash had almost disappeared from the legs, and on the Saturday, it had gone altogether. Her temperature, however, was still 100°, and it continued at that height till Monday, after which it became natural. Her skin was now quite dry and harsh, and there was slight desquamation of the cuticle—that is to say, a separation of the epithelium. This girl affords us a typical case of mild scarlatina.

I will now read to you another case of the same kind. This patient was also a girl, and of the same age.

On a Saturday, she complained of sore-throat; on the next day, the sore-throat continuing, she was very chilly. On the Monday, she had a rash on the face, neck, and chest. On her admission into the hospital these parts were covered with a brilliant scarlet eruption, somewhat elevated and punctiform. On pressing the skin, the redness disappeared, returning when the pressure was removed. The temperature of this girl was also 101.2°, three degrees higher than natural. This was on the third day; for she was taken ill on Saturday, and in these diseases we always reckon the day on which patients were taken ill as the first day. On Tuesday, the fourth day, the rash was less brilliant on the neck, face, and chest, but was very brilliant on the abdomen and legs, again spreading downwards. The tongue was covered with a thin white fur in the centre, but it was red at the tip and edges, and the papillæ were very prominent. The throat was very painful, and the soft palate and the fauces generally were much redder than they should be. The uvula was large and somewhat œdematous—*i. e.*, its loose cellular tissue was the seat of effusion of serum. The tonsils were swollen, but there was no exudation upon them. The temperature was 100°. The glands at the angle of the jaw were large and tender. On the Wednesday, the rash on the legs was darker, more dusky, and more patchy. The temperature was now only 99.8°, still, however, decidedly higher than normal. On Thursday, the rash was almost gone, and desquamation had commenced. The tongue was clean, but red. One tonsil was still swollen, but the child had no soreness of throat. On Friday, the seventh day, the skin of the legs was still dusky, but desquamation was progressing; the abdomen felt rough; the temperature was 98.6°, nearly natural. The child was well. There was no albumen in the urine at any time. The pulse was at first a little more frequent than natural, but in neither of these cases was there any other point which it is important for you to know.

Here, again, is the note of another case, very like those I have related to you, but in one particular a rare case. I have seen several such, and believe they are more common than is supposed. It is a case of relapse; the patient had scarlet fever twice in a short time.

A young woman, twenty years of age, was not quite well on a certain Friday, and on the Saturday, she had sore-throat, and also a symptom which I have not yet mentioned to you, vomiting—sore-throat and vomiting. On the Sunday, Monday, Tuesday, Wednesday, Thursday, and Friday, she was covered with scarlet-fever rash. On the Saturday, the throat was well, and by the Sunday, there was no trace of the rash. On the Sunday following, she was considered quite well. This was May 7th. Now, on Friday, the 19th of May, nearly a fortnight after she was considered quite well, and nineteen days after the disappearance of the rash, she again felt poorly, and complained of frontal headache and nausea. On the Saturday, she had sore-throat, and the same evening the punctiform scarlet-fever rash again came out. On the Monday, there was an abundant rash and sore-throat. On the Tuesday, the rash was growing faint on the trunk, but was fully out on the lower extremities. There was no more complaint of the throat, and the young woman rapidly recovered. As a rule, the second attack runs a short course; but I have seen a patient die in the relapse.

You will note in all these cases, which are simple scarlatina, that the illness came on abruptly; the patient "took ill" at once. In all of them, too, the first symptom was sore-throat. That was the first thing definitely complained of. This is especially the case in adults; but you have here two children, whose first complaint was of their sore-throat. The suddenness with which scarlet fever often commences is well illustrated by the description which a patient once gave me of its commencement. She said: "I was quite well when I went down stairs to fetch some water. Crossing the yard, I felt my throat was sore." That was the first intimation that she was ill. Then rapidly follow the other symptoms.

Another symptom present in one of our cases, and which is very commonly one of the first symptoms, especially among children, is vomiting. Vomiting, when very severe and frequently repeated, is said to indicate that the throat will suffer severely, and certainly I have seen some cases which support this opinion.

Another common symptom which you have heard mentioned is frontal headache. In the whole class of diseases of which scarlet fever is a type—*viz.*, the acute specific diseases, headache is common, and it is nearly always frontal. Why it is so, I don't know. It has nothing to do with the mucous membranes of the frontal sinuses, though it used to be thought that it had. There is the same headache in typhoid fever, which does not specially affect the mucous membrane of the nose or pharynx; the same in typhus, and in all these diseases. Febrile disturbance with frontal headache should at once awaken in your mind the suspicion that it is with one of the diseases of this class that you have to do.

Again, in all these cases there was at the early part of the illness a little sense of chilliness. That is common. Much more rarely the patient has rigors—distinct shiverings—at the onset of the disease.

The temperature at the onset was not taken in any of our cases. Public institutions do not afford an opportunity of taking the temperature at this early period—the first day. But if you do take it then, you will find it very distinctly elevated—100° or 101°, or more; and the higher the elevation on this first day of illness, the more probable it is one of these acute specific diseases.

Those, then, were the first symptoms of illness in the cases I have related. On what day did the rash appear? In one of them there was some redness of the face on the first day, and on the second day the rash was out. In another it did not appear, or we had no evidence that it appeared, until the third day, when the child came into the hospital. The upper part of the abdomen was then, however, covered, so it is probable that it appeared on the preceding day. Thus in each of these two cases it is probable that the true rash came out on the second day. In the case of the young woman, although she was not very well on the Friday, the sore-throat did not begin until the Saturday, and so that was really the first day of the scarlet fever, and her rash was out on the Sunday—the second day. Again, in her next attack she had the sore throat on the Saturday, and on the Monday, she was covered with an abundant rash. Thus in all these cases the rash probably appeared on the second day of the throat affection.

The second day is the common day for the rash to appear in scarlet fever. In the great majority of cases, the patient having sore-throat on the Monday will have the rash out on the Tuesday. Though the second day is the usual date for the appearance of the rash, it is not uncommon for it to be out on the evening of the first day. Now and then it does not come out till the evening of the fourth day, and rarely it does not appear for a week. On any day, then, during the first week the rash may come out. It is said that it sometimes appears later still.

Remember, then, the second day for scarlet fever; the third day for small-pox; the fourth day for measles; the fifth day for typhus; the eighth day for typhoid fever. Those are the typical days. There are frequent deviations, however, in all excepting small-pox, the rash of which very constantly appears on the third day.

The rash in scarlet fever first appears, as it did in these cases, about the neck. Rarely there is a little flush about the face, but the true rash appears about the root of the neck. If you suspect scarlet fever, if the patient is hot and has scarlet lips and a little sore-throat, you throw back the clothes and look for rash at the root of the neck. In measles, you turn back the hair, and look at the margin of the hairy scalp for the first appearance of the rash. In small-pox the eruption appears first about the upper lip, and nose, the centre of the face. In typhoid fever, on the abdomen and thorax. In typhus, the place where the eruption occurs the earliest is often on the backs of the hands, but it occurs also on the trunk.

Note the character of the rash. It was punctiform. This is its character. It comes out in little scarlet dots, which become more and more numerous till they unite, and the whole surface is bright scarlet. The parts which constitute the dots are the most vascular parts of the skin, the papilæ, and the orifices of the follicles, and of the sweat-ducts. The rash is most abundant and darkest where the skin is roughest, on the outer aspect of the limbs. In addition to this scarlet rash, not unfrequently there are a few petechiæ, and that in cases running a perfectly satisfactory course. There are a few extravasations of blood into the substance of the true skin just under the cuticle. These occur most commonly where the skin is the thinnest and most delicate, as at the flexion of the arm. I mention the occurrence of these petechiæ for the benefit of the younger of you, who may think, when first they see a case of scarlet fever with some petechial spots, that the patient is about to be very ill.

So much for the appearance of the rash. Now for its progress. It begins, as I told you, at the root of the neck, and it spreads downwards over the arms and trunk. The parts most affected are usually a little swollen—the hands, for example, are a little puffy. The rash then spreads down to the legs, fading at the upper part of the body, just as it did in the cases I read to you for the purpose of impressing this fact on your memory. It passes down and goes out by the toes, if I may use the expression.

I told you that the rash usually appeared on the second day of the illness. On what day does it disappear? The rash disappears, even from the legs, in a typical case, by the seventh day of illness. You may say that it has a course of about a week from the first day of illness. In our cases it disappeared on the sixth or seventh day of the fever. In some books you are told that the rash disappears earlier, in some later; but you will find the seventh day a convenient day to remember. Other days, all being common, are the sixth, eighth, and ninth. Supposing the patient to have sore-throat on a Monday, the rash would probably be gone by the Monday following. It might disappear on the Sunday, or continue till the Tuesday, or even longer, though that is unusual. Now and then it disappears on the fifth day; now and then it continues till the tenth day, and in some cases it has continued even up to the fourteenth, fifteenth, and sixteenth days. Do not forget this. I have seen such cases myself, and so have most persons who have seen a great many cases.

Desquamation was mentioned as following the disappearance of the rash. Desquamation is the separation of the cuticle, of the epidermis. It commonly begins to separate from the neck, and sometimes will have separated there as a little furfuraceous, branny desquamation, at the time the legs are covered with the rash. Sometimes there will be no desquamation for some days after the disappearance of the rash. It is said usually to occur during or soon after the subsidence of the rash, within a week of its disappearance. Now and then, however, a longer time elapses before desquamation commences.

The patient is often, even in the most simple form of scarlatina, when the rash is at its height, pretty thickly covered with sudamina and minute milliary vesicles. Where these have been present the desquamation commences very early, and is very abundant. It is commonly stated that the desquamation is the throwing off of a poison by the epidermis of the skin. I see, however, no ground for believing this. It seems to me that the desquamation is rather due to a certain degree of effusion beneath the cuticle. With a lens you may often find innumerable vesicles where with your hand and naked eye you could detect none. I believe that the real cause of the desquamation is this subcuticular effusion of serum. We have evidence of the occurrence of effusion into other tissues in the slight swelling of the hands, so common during the disease.

If the desquamation is delayed, it is very common to find the first evidence of it about the roots of the nails. Where the rash was very slight, where perhaps the patient was supposed to have had no rash at all, you may often know that the disease was scarlet fever by that condition. Perhaps one member of a family has had sore-throat, while the others have had scarlet fever; that one was poorly, but you did not think that he had the disease. About a week or ten days after the cessation of the illness, a little opacity of the cuticle round the root of the nails may be noticed, and then a little peeling there. That is almost conclusive evidence that the illness was scarlatina. Do not forget this. It has often served me in good practical stead in determining the nature of an illness.

The desquamation lasts, as a rule, about eight days, or a little longer. From eight to fourteen days is the common period; but occasionally it is a long time before the whole of the cuticle has separated—a month, six weeks, or even two months. The thick cuticle on the soles of the feet may be peeling long after the desquamation on the rest of the body has ceased—when the patient is perfectly well, and has been walking about perhaps for some time. This was strongly impressed on my mind not long since. A gentleman came to me who was invited to stay at the house of a friend. He said, "You know I have had scarlet fever; but it is two months ago. Am I safe from giving the disease to any one else? I ought to tell you that the skin has not yet finished coming off the soles of my feet." I examined his feet, and, finding his statement correct, forbade him going; for I thought he might still communicate the disease.

The desquamation, then, may be so slight as to be perceptible only about the root of the nail, or it may be so considerable as to cover the whole place with dust every time the patient shakes himself.

The lips are always scarlet in the earliest stage of scarlet fever, and this fact often gives an importance to the state of the throat. The tongue, as a rule, is also bright red, especially at the tip and edges. The papillæ are large, and stand out from the thin white fur with which the dorsum of the tongue is covered, just as you heard in the description of one of our typical cases. As the disease progresses the fur disappears, and then (as there was in one of our cases) a bright-red tongue, the epithelium having been thrown off.

The soft palate, and the uvula, and the tonsils are also bright red, the tonsils being swollen. The redness in the early stage is distinctly punctiform, and then the spots coalesce. You may sometimes see that the arches of the palate are vivid red, while there is punctiform redness on the hard palate. The mucous membrane partakes of the eruption of the skin. It often does so in measles likewise; the inside of the lips and the hard palate being covered with eruption identical with that on the skin. I told you that the eruption in measles occurs on the fourth day; but on the second or third day, a day or two before it appears on the skin, the eruption may occasionally be found on the mucous membrane.

In one of our cases it was noted that the glands at the angle of the jaw were swollen and tender. This is according to rule. There is usually a little affection of the glands which receive the absorbents from the soft palate and tonsils. The affection of the throat is the cause of the swelling of the glands. If the throat were not affected the glands would not be swollen. We shall see presently that this is a point of considerable practical importance, and of a good deal of pathological interest. People speak of the scarlet fever process affecting the glands. It is not the scarlet fever process in the ordinary sense of the word—*i. e.*, in the same way that the scarlet fever process affects the soft palate and the skin. The lymphatic glands are affected secondarily to the throat, in consequence of the absorption of something from the surface of the mucous membrane; as much as a bubo in gonorrhœa is the result of the disease of the mucous membrane of the urethra; as much as a bubo in the groin, below Poupart's ligament, after a man has hurt his toe, is the result of the injury to the toe; as much as the swelling of the axillary glands, following a puncture of the finger, is the result of that puncture. You know it is very rare indeed for primary inflammation of the lymphatic glands to occur.

Then the temperature. You notice that it never reached any great height in these cases—100°, 101°. In very severe cases it is often much higher—104°, 105°, 106°.

It is common in cases as mild as these to have a little wandering at night, a little mental disturbance; and very rarely, even in a mild case, on the date of the appearance of the eruption there is an attack of convulsions.

The pulse in our cases never reached 120. It varied from 80 to 100. In scarlet fever the rule is that the pulse rises to a certain degree of frequency, and then goes down from that point. It does not, as in typhoid fever, go up and down, in one part of the day 120, in another 90, and the patient neither better nor worse—*i. e.*, you could not say because his pulse was less frequent, that he was better, or worse because his pulse was more frequent. In scarlet fever the pulse steadily rises, and then, if there is no complication, steadily falls.

In all the cases I related to you the constitutional disturbance was very moderate, and the local specific processes—that is the true scarlatinal processes in the throat and skin—trifling. Bear in mind, however, that in all the scarlet fever group—the acute specific diseases, typhoid, typhus, measles, scarlet fever, small-pox, erysipelas—in all this group there is no necessary relation between the severity of the local specific processes and the severity of the general disease, or between the severity of the general disease and that of the local processes. In our cases they were both mild. If there be a very severe local affection the patient will have a secondary constitutional disturbance due to the local process. If he has a bad sore-throat, he will have a constitutional disturbance dependent on the sore-throat, superadded to the constitutional disturbance of his scarlet fever. But he may have the most intense general disturbance and very little local specific process; the constitutional may be most severe when the local is trifling. The patient may die of the constitutional disturbance when the local process is trifling.

Thus, a man was admitted into this hospital, aged sixty-three. He had been the subject of much mental depression and anxiety. He had for some time lived and slept in the same room and on the same bed with his two children and their mother, all ill with scarlet fever. This man, after he had thus lived and slept in this way for seventeen days, began to complain, on a Sunday, of sore-throat, and on the next day (Monday) he was brought to the hospital insensible. He went in the morning to his place of business, and while there became insensible. When admitted here his respirations were 44; his pulse could not be felt; the surface of the trunk was mottled dusky-red; the outer and posterior aspect of his arms was dusky-red—just the part where the scarlatina eruption occurs and goes most deeply, where the skin is roughest. He rallied about an hour after his admission, so far that his pulse could be counted: it was then 120. His tongue was dry, glazed, and brown. Two hours later there was more duskiness and numerous petechiæ, not merely at a particular part, but over the skin generally. He died at 3 A.M., on Tuesday. On the Sunday, he had sore-throat; on the Monday, he was taken at once with the active poison; and on the Tuesday, at three o'clock in the morning, he was dead. After death, what did we find? Numerous subserous and submucous petechial spots under the endocardium, under the pericardium, under the pleura, under the peritonum, under the mucous membrane of his stomach, under the mucous membrane of his bowel—everywhere small extravasations of blood. His tonsils were large; there was vascularity of the pharynx; and his spleen was enlarged. That is one of the features of scarlatina; as it is of all these diseases—typhoid, typhus, measles, small-pox, scarlet fever, erysipelas: in all there is softening and enlargement of the spleen.

We examined this man most carefully. I have here long notes of the state of every organ in his body, the microscopic as well as the naked eye appearances of each; but we failed to discover anything more than I have mentioned to you. He died from the poison of scarlet fever. There was no lesion of the heart, brain, lungs, or kidneys, to account for his death; he died from the general condition.

Again, a young woman, aged twenty-one, had been ailing for some time with some soreness of throat, sometimes better, sometimes worse; but she had been subject to sore-throats, and not much importance was attached to this particular attack. On Monday, June 10th, she felt ill, and had increased soreness of throat, but was still able to go out. She passed a restless night and was decidedly ill on the morning of the next day (Tuesday). On the Tuesday night, she was very bad, tossing about, but not delirious. On Wednesday, however, she was violently delirious; several persons were required to keep her in bed. All that night she was violently delirious. On Thursday morning, she was insensible; and on Thursday afternoon, I saw her. She was then lying on her back, inclined to the right side, resisting all efforts to move her. Her conjunctivæ were considerably injected, as they are in all these cases. It seems as if the capillary circulation generally were in some way interfered with; all the vessels are loaded with blood and very little circulation going on. Her pupils acted but little. She muttered incoherently, but was quite unable to be aroused so as to exhibit consciousness. She passed urine into the bed freely. There was a thin sero-purulent discharge from the nose and ears. There was no swelling of the glands. She had a dusky-scarlet rash on the trunk and upper extremities; none on the lower. Her pulse was 180, very weak. Subsequently she became again violently delirious, and died at 10:30 P.M. She was taken ill on the Monday, and died on the Thursday night. At the *post mortem* nothing more was found than in the last case.

Another case of the same kind. H. B——, a very fine young man, six feet high, and broad and large in proportion. We could get no history of his illness, but he came under observation on October 13th. He had had no sleep on the preceding night, and when I saw him he was delirious, leaving his bed and wandering about the ward. He said that he had been in the hospital four days. Note that in the whole group of these diseases there is a tendency on the part of the patient to lengthen time. He nearly always thinks it a longer time since you saw him last than it really is. Everything seems long to him. It is not so in some other acute diseases—in pneumonia, for example; but in this class of diseases—the acute specific—although the patient answers you quite rationally, and tells you correctly the day on which he came into the hospital, he thinks he has been in a much longer time than he really has.

The expression of this man's face was natural. He had no headache. He was able to leave his bed alone and easily. His tongue was moist and furred, not particularly red. He complained of some soreness of the throat, but swallowed without any difficulty. He had no cough. His skin was hot and moist, and covered over the chest and abdomen with a bright scarlet rash, which disappeared on pressure. On the arms, legs, back, and beneath the clavicle, the rash was more dusky; and, after pressure, minute purple spots (petechiæ) were perceptible over the whole of the body. On the night of the 13th he was violently delerious; a man had to be with him to keep him in bed. He was restrained with difficulty; bit and struggled with great violence. At five in the morning, he became quieter, his breathing became difficult about six, and at half-past eight he died.

On the 13th, the rash had not spread to his legs, so that we may suppose that he had not been ill above four days. His *post mortem* examination was made, and, as he died in the hospital, it was made very carefully. The notes of it which I have here, occupy eight closely-written quarto pages. I am not going to read them to you, for all that we found was just what we found in the last case—petechial spots under the mucous membrane of the stomach and the bowels, under the endocardium, under the pericardium, under the pleura. There was a little recent lymph at the base of the lung, probably of only a few hours' duration, and a spleen which weighed a pound and a half. There was a little congestion of his lung, such as might have resulted from his struggles; but beyond these appearances we found nothing.

Here is another case. A young man came under my care with a very little sore-throat, but covered with the rash, and with considerable febrile disturbance. After a violent fit of delirium, he sank exhausted, with a frequent, feeble pulse. He died in half an hour from the time he was running furiously about the ward. After death the appearances we found were precisely what I have described to you—enlarged spleen, subserous and submucous petechial spots, and that was all. In these cases of death from the general disease, you will have observed that in several there was a full, free, and even bright rash.

Sometimes, in place of the rash being bright red it is more dusky. I told you it was dusky over part of the skin in one of the cases I have described. Sometimes there is more congestion of the lung. Sometimes, instead of violent delirium, there is muttering delirium. The girl, I told you, had muttering delirium, and then became violent again. Thus there may be extreme prostration instead of active delirium, or active delirium followed by extreme prostration. I have no account of the temperature in these cases, but it has been taken in similar cases, and has been found, it is said, to be high, very high; not, however, equal, so far as I am aware, to that which has been met with in acute rheumatism, but 104°, 105°, 106°. Some have supposed that in these cases the death is due to the high temperature. I am doubtful of that myself, in scarlatina. I think the death is due to the changed condition of blood. It is evidently very much changed, from the occurrence of the petechiæ; and the change in the tissues is dependent on the change in the blood. More observations on the temperature in these cases are required before its influence can be clearly admtited.

There are two other forms of scarlet fever, where there is only the general affection, to which you ought to have your attention directed; both are very mild forms. One of these is scarlatina without inflammation of the throat—*scarlatina sine angina.* I doubt its existence; that is all I can say about it. There are cases without soreness of throat, but if you look into the throat, in all the cases I have seen, there is distinct redness and swelling of the tonsils, at any rate—considerable abnormal vascularity of the fauces and pharynx. I have often known patients to say they had no sore-throat, but when I have looked I have always seen abnormal vascularity. So I do not believe in scarlatina without any throat affection.

The other form is scarlatina without any eruption. About this, too, I have some doubts. Of the other I speak from having seen a large number of cases in which the patients have vowed that they had no throat affection at all, and yet I saw it. The subjective symptoms were absent, the objective were present. But it is difficult to prove the absence of the eruption. Certainly people sometimes have the disease with a very little and a very transient eruption, just a little scarlet blush, lasting a few hours, and probably there are some cases in which there is none.

Thus I have described to you cases of that form of scarlet fever in which the disease is the poisoned state of the system only, and I have described cases illustrating varieties of this form of scarlet fever, from the mildest to the gravest. In no case mentioned to-day was there any grave local disease—nothing complicating the general disease. What is the treatment of uncomplicated scarlet fever, with trifling local specific process? For scarlet fever proper, for the poisoned condition of the system, so far as I know, we have no remedy. There are those who say ammonia is the remedy; there are those who say that hydrochloric acid is the remedy; and so on with a variety of drugs. But at the present moment my experience tells me that we have no remedy for the general disease. We can only act upon the broadest general principles of calming the patient when excited; of stimulating him, to keep his heart beating, when he is exceedingly weak; of cooling the surface when the heat is excessive. Give him pure, fresh air to breathe. It is a disease that runs a definite course, and if you can keep the sufferers alive for a certain time they get well from that general disease which killed some of our patients. So you watch for the symptoms which threaten death, and endeavor to avert them. The *very* grave cases are hopeless from the first. For the mild cases little treatment is required. A cool room, light clothes, unstimulating diet, a mild aperient, a little chlorate of potash, which seems to allay the irritation or inflammation of the throat, and that is all you need do. Some give a little dilute mineral acid; sulphuric acid was all that used to be employed at the Fever Hospital when I was there. I think myself that the best is the chlorate of potash drink. I put a dram of the salt into a pint of barley-water, and let the patient sip it down as he pleases.

You will see that it is very different when I come to speak of the complications of scarlet fever. For some of the aggravations of the acute specific process, for some of the throat affections, we may do a great deal, and there we often save life directly; but for the disease itself, that which is the root of the matter, I repeat, so far as I know, you can do nothing. By the chlorate of potash you may do something toward allaying the throat affection; by a mild aperient you may allay a little the febrile disturbance which would be increased by confined bowels; by keeping the room well ventilated you prevent any increase of fever from the room becoming too close, and its air impure. And all the time you must watch the state of the urine, the skin, and the throat. One point I desire to impress on you—namely, the great importance of changing the clothes of the patient, as well as the air of the room. The blankets of the bed should be changed frequently, as well as the body-linen and sheets.—*Lancet, Jan. 8th, 1870.*

